# Kinetics of Flavoenzyme-Catalyzed Reduction of Tirapazamine Derivatives: Implications for Their Prooxidant Cytotoxicity

**DOI:** 10.3390/ijms20184602

**Published:** 2019-09-17

**Authors:** Aušra Nemeikaitė-Čėnienė, Jonas Šarlauskas, Violeta Jonušienė, Audronė Marozienė, Lina Misevičienė, Aliaksei V. Yantsevich, Narimantas Čėnas

**Affiliations:** 1 State Research Institute Center for Innovative Medicine, Santariškių St. 5, LT-08406 Vilnius, Lithuania; ausra.ceniene@imcentras.lt; 2 Department of Xenobiotics Biochemistry, Institute of Biochemistry of Vilnius University, Saulėtekio 7, LT-10257 Vilnius, Lithuania; jonas.sarlauskas@bchi.vu.lt (J.Š.); audrone.maroziene@bchi.vu.lt (A.M.); lina.miseviciene@bchi.vu.lt (L.M.); 3 Department of Biochemistry and Molecular Biology, Institute of Biosciences of Vilnius University, Saulėtekio 7, LT-10257 Vilnius, Lithuania; violeta.jonusiene@gf.vu.lt; 4 Institute of Bioorganic Chemistry, NAS of Belarus, Kuprevicha 5/2, BY-220072 Minsk, Belarus; al.yantsevich@gmail.com

**Keywords:** tirapazamine, reductive activation, oxidative stress, cytotoxicity

## Abstract

Derivatives of tirapazamine and other heteroaromatic *N-*oxides (ArN→O) exhibit promising antibacterial, antiprotozoal, and tumoricidal activities. Their action is typically attributed to bioreductive activation and free radical generation. In this work, we aimed to clarify the mechanism(s) of aerobic mammalian cell cytotoxicity of ArN→O performing the parallel studies of their reactions with NADPH:cytochrome P-450 reductase (P-450R), adrenodoxin reductase/adrenodoxin (ADR/ADX), and NAD(P)H:quinone oxidoreductase (NQO1); we found that in P-450R and ADR/ADX-catalyzed single-electron reduction, the reactivity of ArN→O (*n* = 9) increased with their single-electron reduction midpoint potential (*E*^1^_7_), and correlated with the reactivity of quinones. NQO1 reduced ArN→O at low rates with concomitant superoxide production. The cytotoxicity of ArN→O in murine hepatoma MH22a and human colon adenocarcinoma HCT-116 cells increased with their *E*^1^_7_, being systematically higher than that of quinones. The cytotoxicity of both groups of compounds was prooxidant. Inhibitor of NQO1, dicoumarol, and inhibitors of cytochromes P-450 α-naphthoflavone, isoniazid and miconazole statistically significantly (*p* < 0.02) decreased the toxicity of ArN→O, and potentiated the cytotoxicity of quinones. One may conclude that in spite of similar enzymatic redox cycling rates, the cytotoxicity of ArN→O is higher than that of quinones. This is partly attributed to ArN→O activation by NQO1 and cytochromes P-450. A possible additional factor in the aerobic cytotoxicity of ArN→O is their reductive activation in oxygen-poor cell compartments, leading to the formation of DNA-damaging species similar to those forming under hypoxia.

## 1. Introduction

**Scheme 1:**
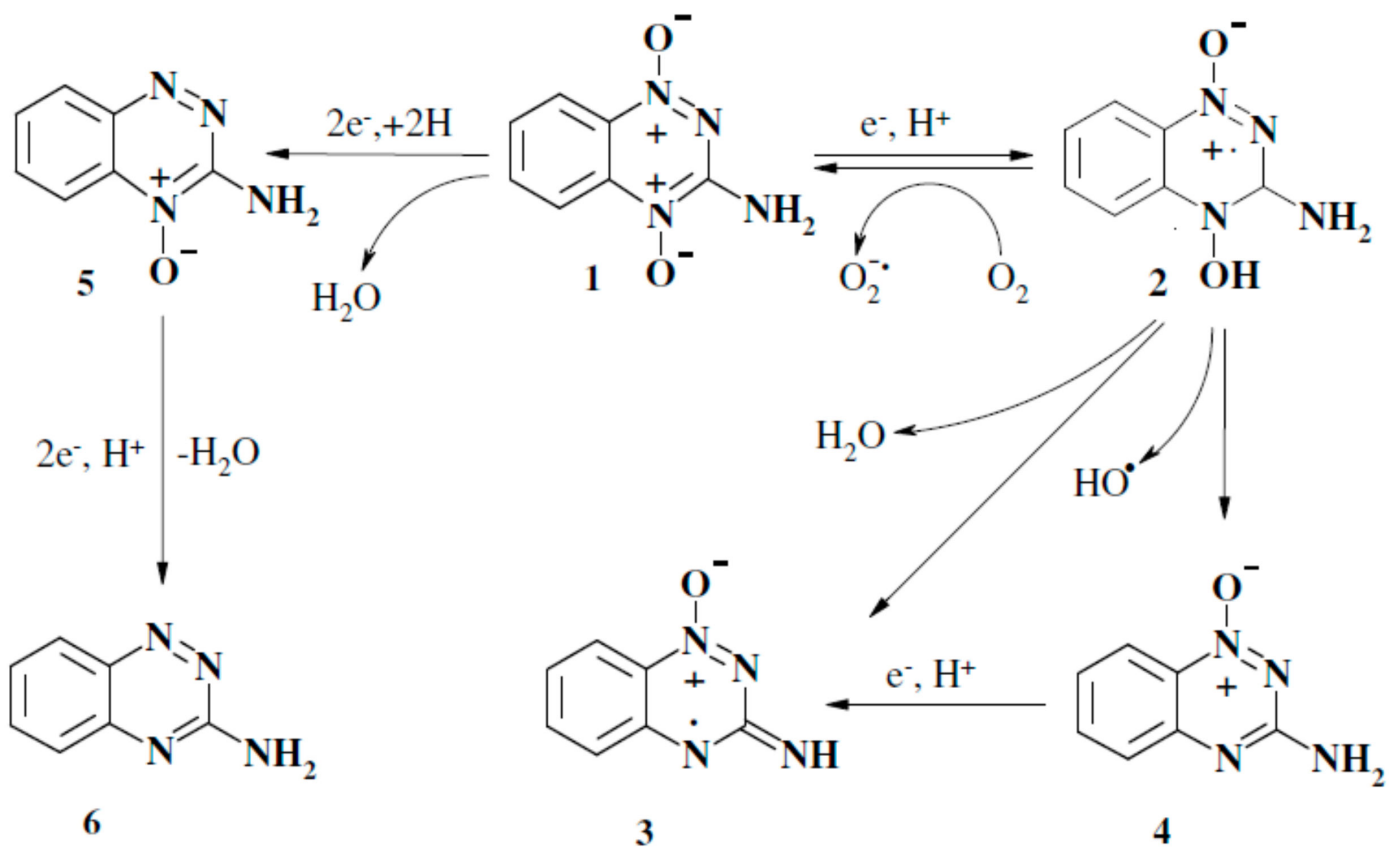
Pathways of the reduction of tirapazamine in the cell.

*N-*oxides of 1,2,4-benzotriazine and quinoxaline (ArN→O) frequently possess promising antibacterial, antiprotozoal, and tumoricidal activities, including their potential application in the treatment of hypoxic tumors ([1,2], and references therein). In most cases, their action is attributed to bioreductive activation and free radical generation. Among their representatives, the redox reactions of 3-amino-1,2,4-benzotriazine-1,4-dioxide (tirapazamine, TPZ) and its derivatives have been studied most comprehensively. TPZ (**1**) is enzymatically reduced in a single-electron way to a free radical (**2**), which forms DNA-damaging species under hypoxic conditions, namely, an oxidizing hydroxyl radical (OH^.^) ([3], and references therein), and/or a highly reactive benzotriazinyl radical (**3**) that abstracts a hydrogen atom from DNA ([2,4,5], and references therein) (Scheme 1). The nature of DNA-damaging species is still a matter of debate.

In the cell, the final relatively nontoxic metabolites of TPZ are its mono-*N*-oxide (**4**), formed possibly via free radical (**2**) intermediate [2,3], and its nor-oxide (**6**), presumably formed via 4-*N-*oxide (**5**) intermediate [6]. Their formation is strongly inhibited under oxic conditions. The single-electron reductive activation of TPZ under hypoxic and oxic conditions is carried out mainly by microsomal NADPH:cytochrome P-450 reductase (P-450R) ([7], and references therein), and/or by insufficiently characterized intranuclear NAD(P)H-oxidizing flavoenzymes [8]. Flavoenzyme NAD(P)H:quinone oxidoreductase (DT-diaphorase, NQO1) also reduces TPZ into compounds (**4**) and (**6**), however, it is unclear whether it is involved in TPZ cytotoxicity [9,10,11]. The data about the involvement of cytochromes P-450 (P-450) in the reductive metabolism and cytotoxic action of TPZ are also controversial [11,12,13,14,15,16,17].

The aerobic cytotoxicity of TPZ and other aromatic *N-*oxides is attributed to their redox cycling (Scheme 1), leading to the formation of superoxide (O_2_^−·^) and subsequent oxidative stress [1,2]. Because their aerobic cytotoxicity is lower than under hypoxia, it attracted relatively less attention ([18,19,20]. On the other hand, some TPZ analogues possess anticancer activity at micromolar concentrations even under oxic conditions [21,22]. Besides, the redox cycling events also may be important in the antimicrobial and antiparasitic action of TPZ analogs ([2,23,24,25], and references therein). However, the kinetic data on the enzymatic reduction of ArN→O are scarce and scattered, and their relationship with aerobic cytotoxicity of ArN→O is understood incomprehensively.

The aim of this work was to characterize the relation between the aerobic cytotoxicity of TPZ derivatives and their reactivity towards single-electron transferring P-450R, and another model system, adrenodoxin reductase/adrenodoxin (ADR/ADX). An ‘outer-sphere’ electron transfer model [26] was applied for the analysis of kinetic data. Taken together with further cytotoxicity studies, it revealed that the cytotoxicity of ArN→O was higher than that of quinones with similar redox properties. We found that this phenomenon is partly attributed to the action of NQO1 and P-450.

## 2. Results

### 2.1. Enzymatic Single-Electron Reduction of Aromatic N-Oxides

**Figure 1:**
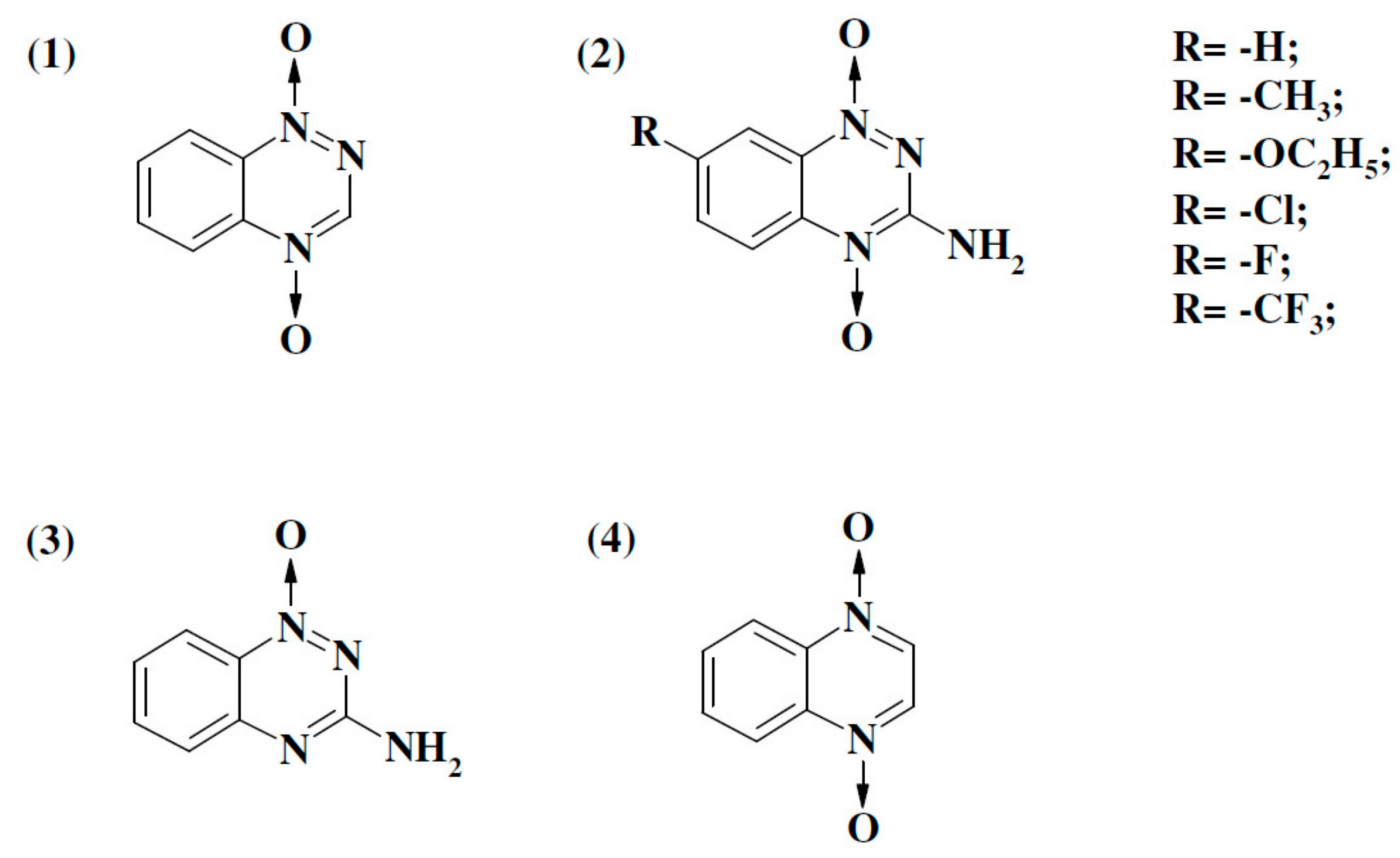
Formulae of aromatic *N*-oxides used in this work: 1,2,4-benzotriazine-1,4-dioxide (**1**), 3-amino-1,2,4-benzotriazine-1,4-dioxide (TPZ) and its derivatives (**2**), 3-amino-1,2,4-benzotriazine-1-oxide (**3**), and quinoxaline-1,4-dioxide (**4**).

In this work, we used a number of aromatic *N-*oxides, whose single-electron reduction midpoint potentials (*E*^1^_7_) vary between –0.318 V and –0.575 V [4,18,27] (Figure 1). We studied their reactions with P-450R, which plays the most important role in the single-electron reduction and redox cycling of quinones, nitroaromatics and, possibly, ArN→O in the mammalian cell [7]. As an additional model reaction, we studied the reduction of ArN→O by Fe_2_S_2_ redox protein adrenodoxin (ADX). Flavoenzyme NADPH:adrenodoxin reductase (ADR) reduces quinones and nitroaromatics in single-electron way, however, these reactions are relatively slow and complicated by the NADPH substrate inhibition [28,29]. On the other hand, ADX stimulates quinone- and nitroreductase activity of ADR, eliminating the inhibition by NADPH and providing an alternative more efficient electron-transfer pathway via ADX [28,29]. The studies of ArN→O reduction by ADX are also important in view of possible but insufficiently characterized role of FeS redox proteins in the reductive activation of prooxidant drugs and xenobiotics.

The bimolecular rate constants (*k*_cat_/*K*_m_) of reduction of ArN→O by P450R and ADR/ ADX are given in Table 1. For the most active oxidants of P-450R, 7-CF_3_-, 7-F-, and 7-Cl-substituted TPZ, and 1,2,4-benzotriazine-1,4-dioxide (Table 1), the *k*_cat_ at their saturating concentrations were in the range of 17.0–19.0 s^−1^, i.e., close to 50% of the rate of cytochrome *c* reduction by P-450R. In other cases, the reaction rates were proportional to the concentration of compounds up to the limits of their solubility, ≥600 µM. The *k*_cat_ of ArN→O in ADR/ADX-catalyzed reactions were in the range of 3.7-3.3 s^−1^, which again was close to 50% of ADX- mediated cytochrome *c* reduction rate. In both P450R- and ADR/ADX-catalyzed reactions, ArN→O undergo redox cycling, e.g., 100 µM TPZ or its 7-CF_3–_ or 7-Cl– derivatives oxidized significant excess NADPH, 300 µM, and the NADPH oxidation was accompanied by O_2_ consumption proceeding with similar rate.

The increase of the reactivity of heteroaromatic *N-*oxides with their *E*^1^_7_ values (Table 1) may be related to an ‘outer-sphere’ electron transfer mechanism [26]. In this case, the rate constant of single-electron transfer between reagents (*k*_12_) depends on the electron self-exchange rate constants of reagents (*k*_11_ and *k*_22_) and equilibrium constant of the reaction (*K*) (log *K* = ∆*E*^1^ (V)/0.059, where ∆*E*^1^ is the difference in the standard single-electron transfer midpoint potential of reactants):

*k*_12_ = (*k*_11_ × *k*_22_ × *K* × *f*)^1/2^ (1)

and

log *f* = (log *K*)^2^/4 log (*k*_11_ × *k*_22_/Z^2^ (2)

where Z is a frequency factor (10^11^ M^−1^·s^−1^). In the reaction of electron donor with a series of homologous electron acceptors (*k*_22_ = constant), the Equation (1,2) predict a parabolic (square) dependence of log *k*_12_ on ∆*E*^1^ with a slope ∆log *k*_12_/∆∆*E*^1^ = 8.45 V^−1^ at ∆*E*^1^ = ±0.15 V.

**Figure 2:**
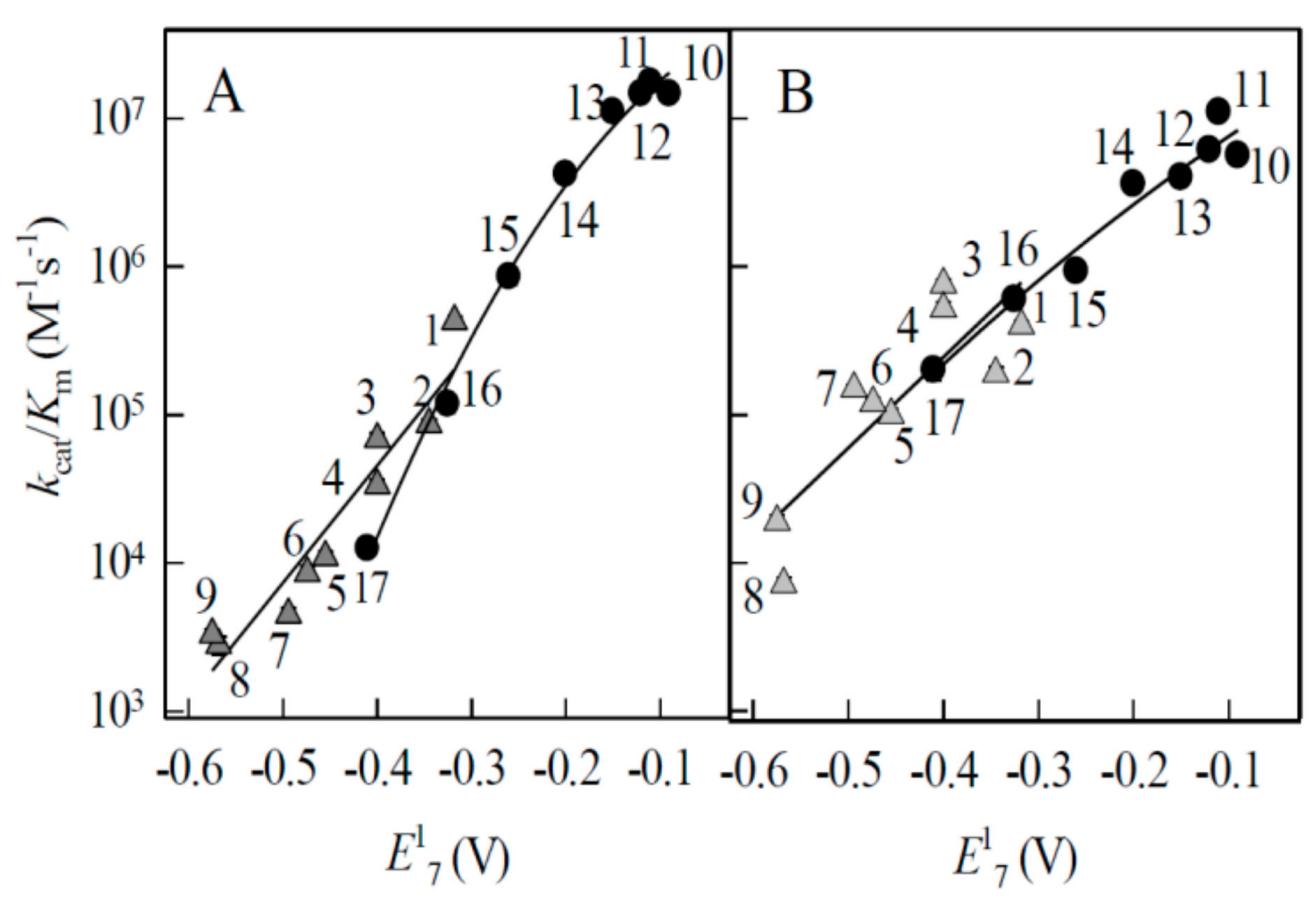
Dependences of bimolecular rate constants of enzymatic single-electron reduction of ArN→O and quinones on their single-electron reduction midpoint potential. (**A**) The reactivity of ArN→0 (triangles) and quinones (circles) in P-450R-catalyzed reactions. (**B**) The reactivity of ArN→0 (triangles) and quinones (circles) in ADR/ADX-catalyzed reactions. The numbers of compounds correspond to those in Table 1. The reactivity of ArN→0 and quinones are described by the first order and second order polynomial regressions, respectively.

This type of dependences frequently describes the single-electron reduction of quinones and nitroaromatics by flavoenzymes ([31,32], and references therein). To the best of our knowledge, the *k*_22_ values for aromatic *N-*oxides have not been experimentally determined. However, the value of log *k*_22_ = 8.59 may be calculated using log *k*_11_ = 2.65 for O_2_/O_2_^-.^ couple [33] from the rate constants of oxidation of quinoxaline-1,4-dioxide radicals with oxygen [5] (Appendix A). This shows that aromatic *N-*oxides may possess high electron self-exchange rate which is close to that of another group of redox cycling compounds, quinones (*k*_22_ ~10^8^ M^−1^·s^−1^ [1]). This was experimentally confirmed by the determination of the rate constants of reduction of a series of quinones by P450R and ADR/ADX (Table 1). In both systems, ArN→O and quinones followed very close log *k*_cat_/*K*_m_ vs. *E*^1^_7_ relationships (Figure 2A,B). For this reason, quinones were used as the reference compounds in further cytotoxicity studies.

### 2.2. Studies of NQO1-Catalyzed Reduction of Aromatic N-Oxides

Flavoenzyme NQO1 performs rapid NAD(P)H-dependent two-electron reduction of quinones into their hydroquinones ([34], and references therein). The reduction rate constants of quinones used in this work (Table 1) were determined previously [34]. Their *k*_cat_ vary from 0.5 s^−1^ (1,8-dihydroxy-9,10-anthraquinone) to 10^3^ s^−1^ (tetramethyl–1,4-benzoquinone), and *k*_cat_/*K*_m_ vary from 1.2 × 10^6^ M^−1^·s^−1^ (1,8-dihydroxy-9,10-anthraquinone) to 5.4 × 10^8^ M^−1^·s^−1^ (1,4-naphthoquinone). Apart from the reduction potential, the specificity of NQO1 strongly depends on the structure of oxidants, whose requirements are incompletely characterized at this time [34,35,36]. For example, another group of prooxidant compounds, nitroaromatics, are reduced 10^2^–10^5^ times slower than quinones ([35], and references therein). In line with previous observations [9], heteroaromatic *N*-oxides used in this work were slow substrates for NQO1 (Table 2). The control experiments using NADPH regeneration system demonstrated that the reduction of ArN→O was responsible for not more than 5–10% of total 340 nm absorbance changes in the reaction course. Thus, NADPH oxidation rates measured at 340 nm could be taken as accurate within these limits.

**Figure 3:**
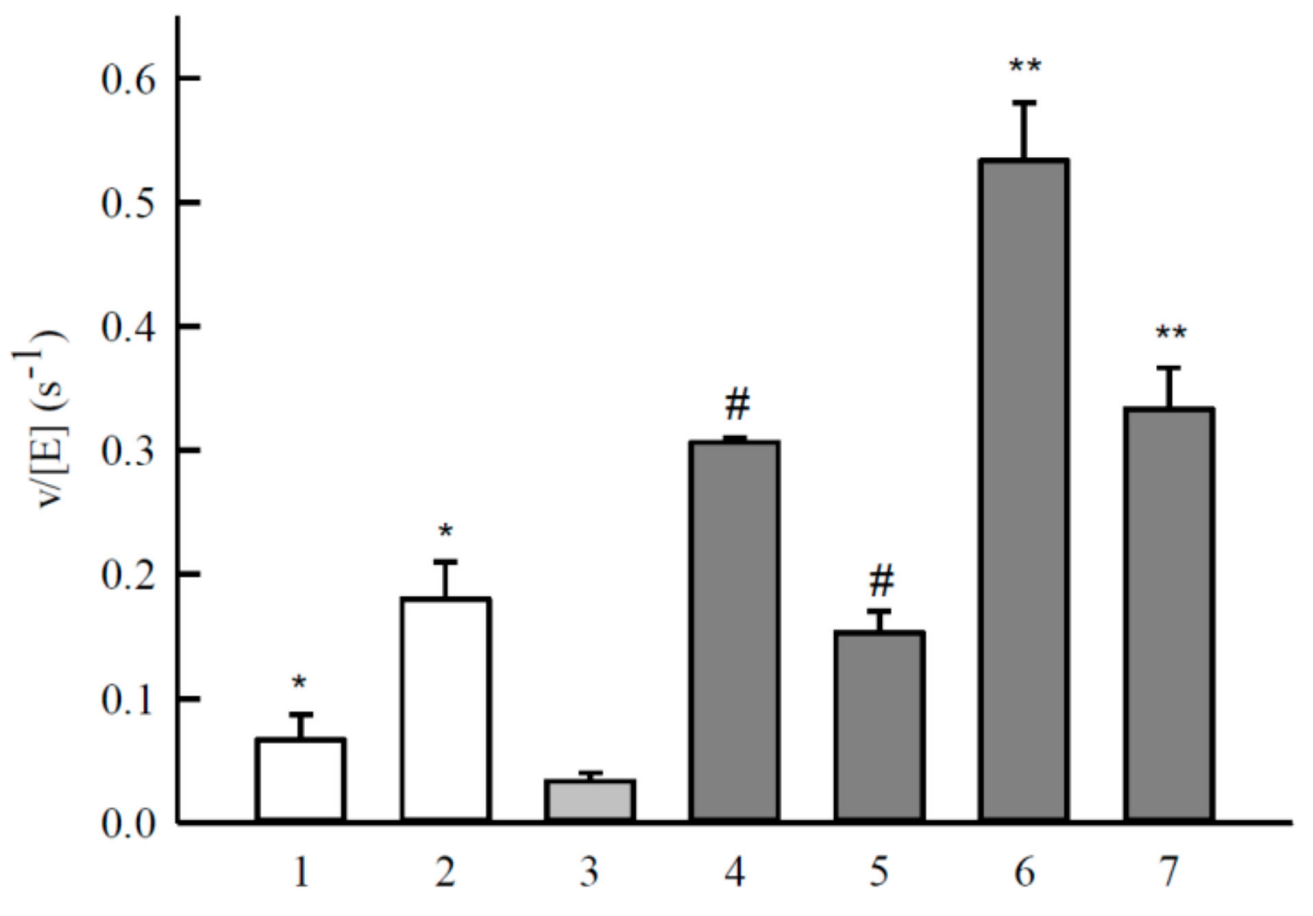
NAD(P)H:quinone oxidoreductase (NQO1)-catalyzed redox cycling of TPZ. The rates of NQO1-catalyzed oxidation of NADPH (1,2), depletion of TPZ (3), and reduction of cytochrome *c* (4-7) in the presence of 300 µM NADPH. Additions: NADPH (1), NADPH + 300 µM TPZ (2,3), NADPH + 50 µM cytochrome *c* (4), NADPH +cytochrome *c* + 100 U/mL SOD (5), NADPH + TPZ + cytochrome *c* (6), NADPH + TPZ + cytochrome *c* + SOD (7), *n* = 3, *p* < 0.02 for 1 against 2 (*), for 4 against 5 (#), and 6 against 7 (**).

The *k*_cat_ for TPZ reduction (Table 2) expressed in moles NADPH oxidized per mole of enzyme per second, was close to previously reported value [8]. The rate of TPZ disappearance was equal to 20–25% NADPH oxidation rate (Figure 3). In parallel, TPZ stimulated NADPH:cytochrome *c* reductase activity of NQO1, which was partly inhibited by superoxide dismutase (Figure 3). For comparison, the rates of oxidation of NADPH and reduction of cytochrome *c* by NQO1 in the presence of 50 µM tetramethyl-1,4-benzoquinone were equal to 720 ± 30 s^−1^ and 1400 ± 52 s^−1^, respectively. In the presence of superoxide dismutase, the rate of reduction of cytochrome *c* was almost unchanged, 1415 ± 60 s^−1^.

### 2.3. Studies of Cytotoxicity of Aromatic N-oxides

In cytotoxicity studies, we determined the concentrations of ArN→O for 50% cell survival (cL_50_) in murine hepatoma MH22a cells, and, in some cases, their concentrations for 50% of maximal inhibition (GI_50_) of proliferation of human colon adenocarcinoma HCT-116 cells (Table 3).

In addition, the same cytotoxicity parameters for a number of quinones were determined (Table 3).

**Figure 4:**
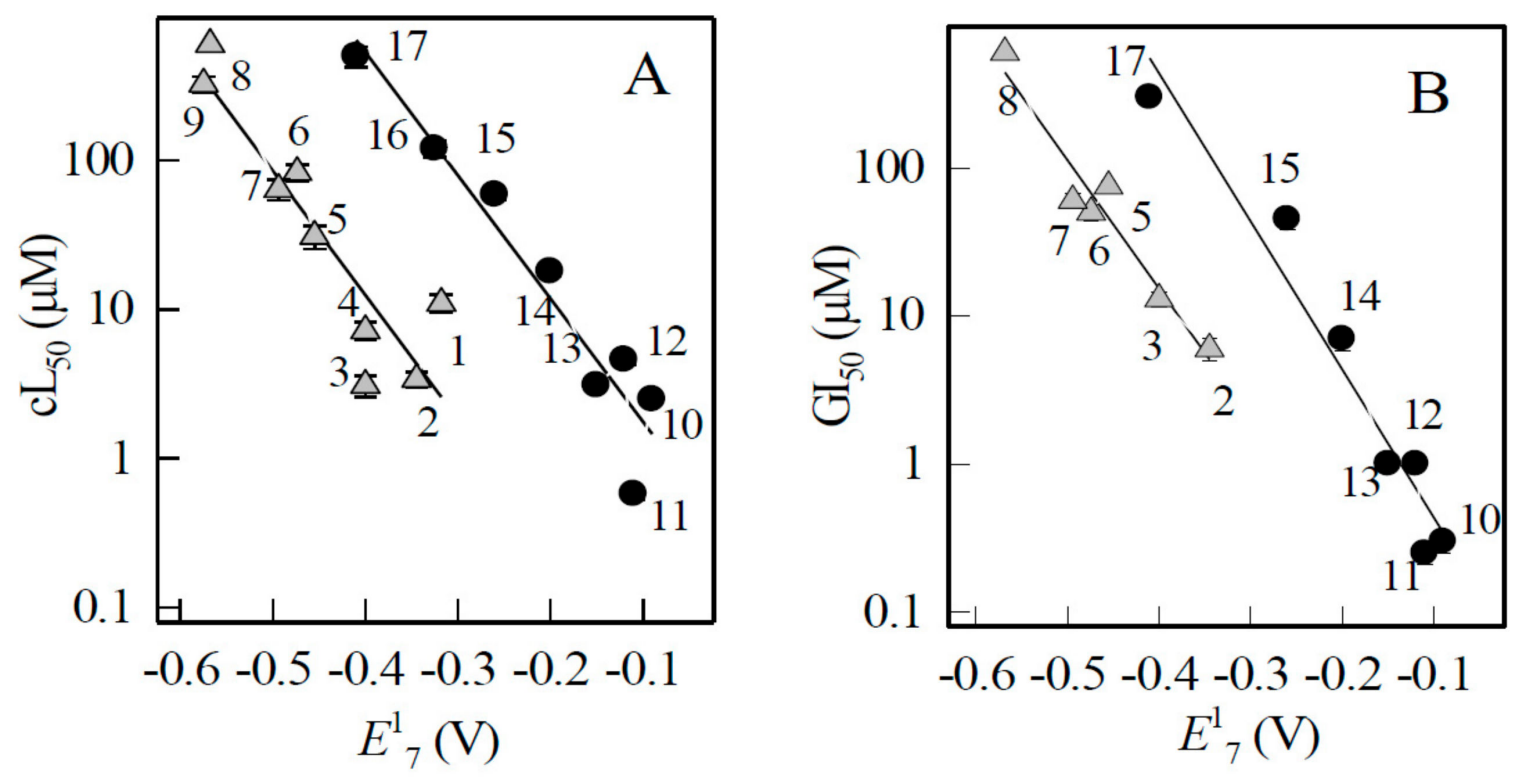
Dependence of cytotoxicity and growth inhibition potency of ArN→O and quinones on their single-electron reduction midpoint potential. (**A**) Cytotoxicity of ArN→O (triangles) and quinones (circles) in MH22a cells. (**B**) Growth inhibition potency of ArN→O (triangles) and quinones (circles) HCT-116 cells. The numbers of compounds correspond to those in Table 1. The cytotoxicity and growth inhibition potency of ArN→0 and quinones is described by the first order polynomial regressions.

We found that the cytotoxicity of ArN→O and quinones in both cell lines increased with their *E*^1^_7_, and that ArN→O were typically by one order of magnitude more cytotoxic than quinones (Figure 4).

**Figure 5:**
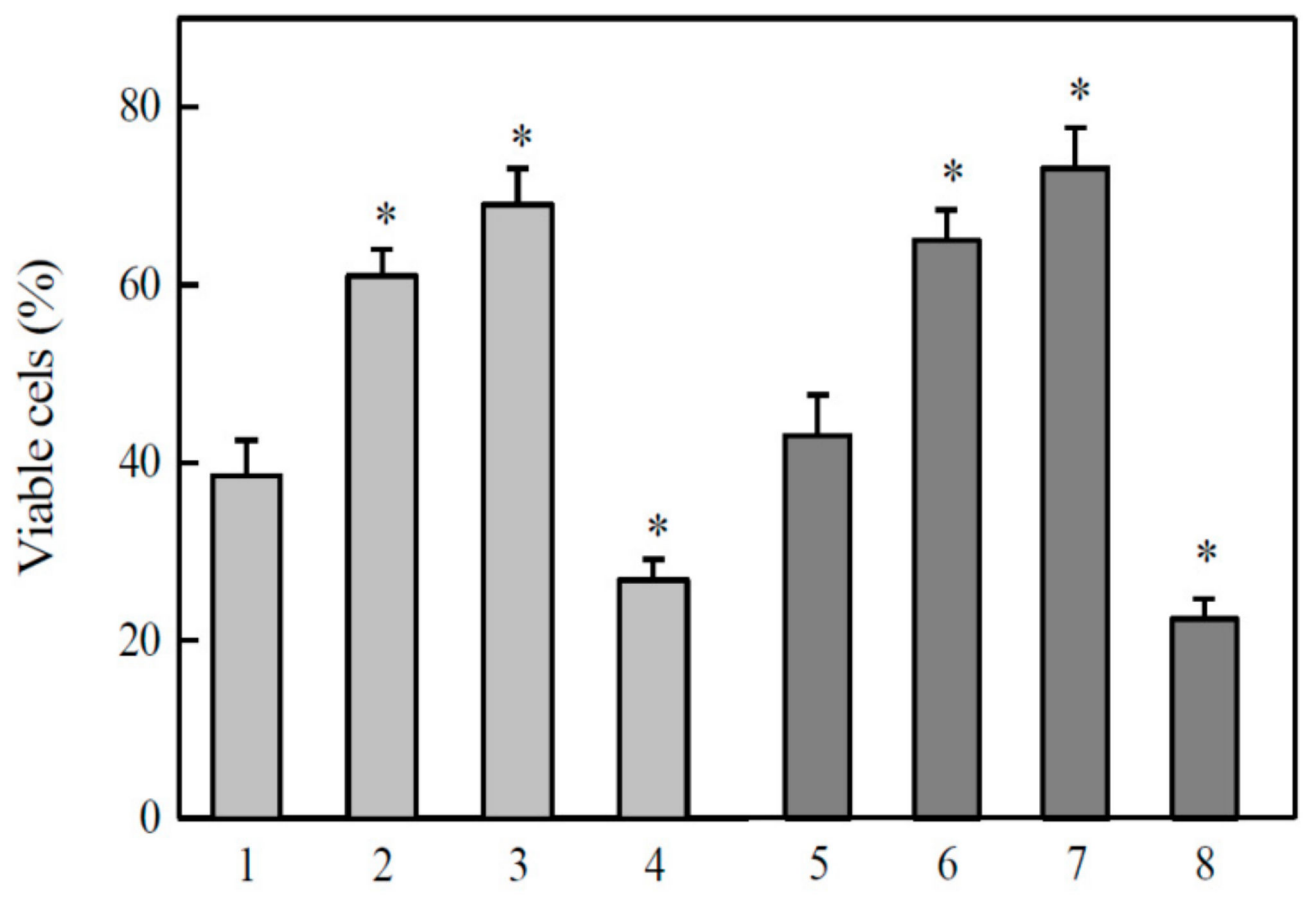
Effects of antioxidants and 1,3-bis(2-chloroethyl)-1-nitrosourea (BCNU) on the cytotoxicity of TPZ and 9,10-phenanthrene quinone in MH22a cells. 40 µM TPZ (1–4), 5.0 µM 9,10-phenanthrene quinone (5–8), additions: none (1,5), + 300 µM desferrioxamine (2,6), + 3.0 µM N,N’-diphenyl-p-phenylene diamine (DPPD) (3,7), + 20 µM BCNU (4,8). Additions of DPPD, desferrioxamine and BCNU did not affect the cell viability. *n =* 3, *p <* 0.02 for 1 against 2–4, and for 5 against 6–8 (*). Cell viability in control experiments, 98.5 ± 1.0%.

The linear negative dependence of log cL_50_ on *E*^1^_7_ of quinones or nitroaromatics shows that the main factor of their cytotoxicity is redox cycling and oxidative stress ([38,39,40], and references therein), because, as a rule, the rates of single-electron reduction of these compounds increase with *E*^1^_7_ of oxidant [31,32]. In accordance with this, the cytotoxicity of tirapazamine and 9,10-phenanthrene quinone in MH22a cells was decreased by desferrioxamine and the antioxidant *N,N’-*diphenyl-*p-*phenylene diamine (DPPD), and enhanced by 1,3-bis(2-chloroethyl)-1-nitrosourea (BCNU), the latter inactivating glutathione reductase and depleting reduced glutathione [41] (Figure 5). The same effects of the above compounds were observed in the cytotoxicity studies of other randomly selected quinones and ArN→O.

**Figure 6:**
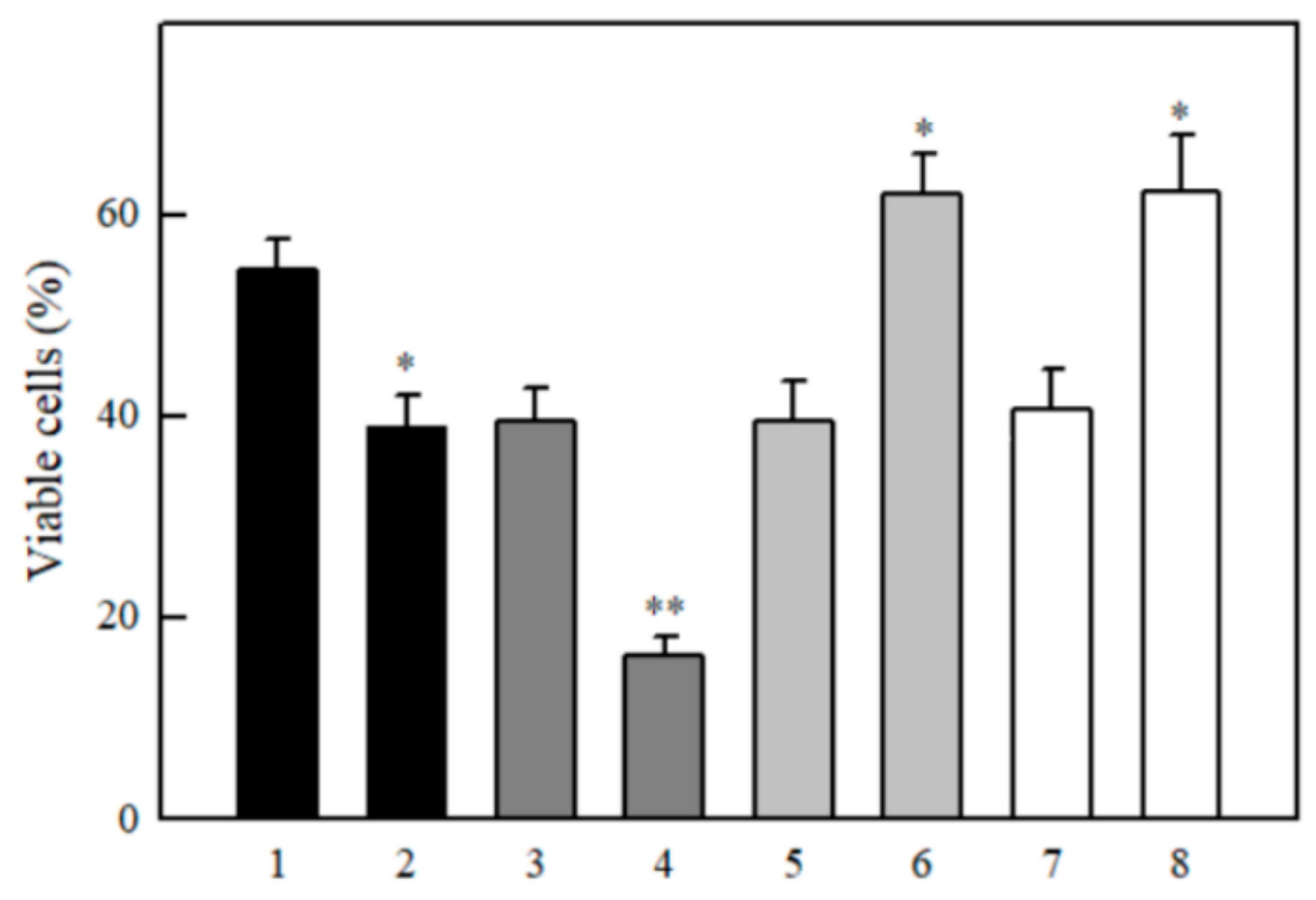
Effects of dicoumarol on the cytotoxicity of quinones and ArN→O in MH22a cells. 50 µM tetramethyl-1,4-benzoquinone (1,2), 5.0 µM 9,10-phenanthrene quinone (3,4), 50 µM TPZ (5,6), 10 µM 7-F-TPZ (7,8) in the absence (1,3,5,7) and in the presence of 20 µM dicoumarol (2,4,6,8). This dicoumarol concentration did not affect the cell viability in control experiments, 98.5–99.3%. *n* = 3, *p* < 0.02 for 1 against 2, 5 against 6, and 7 against 8 (*), *p* < 0.01 for 3 against 4 (**).

The enhanced cytotoxicity of ArN→O is not related to their high lipophilicity. In general, the calculated octanol/water distribution coefficients at pH 7.0 (log *D*) of ArN→O are lower than those of quinones (Table 3). Concerning other enzymatic mechanisms possibly enhancing the cytotoxicity of ArN→O, the involvement of NQO1 and cytochromes P-450 is a matter of debate [9,10,11,12,13,14,15,16,17]. Here we studied the effects of NQO1 inhibitor, dicoumarol, and several inhibitors of cytochromes P-450 on the cytotoxicity of ArN→O and quinones. Dicoumarol potentiated the cytotoxicity of tetramethyl-1,4-benzoquinone and 9,10-phenanthrene quinone (Figure 6). On the other hand, it decreased the cytotoxicity of tirapazamine and its 7-F-derivative (Figure 6).

**Figure 7:**
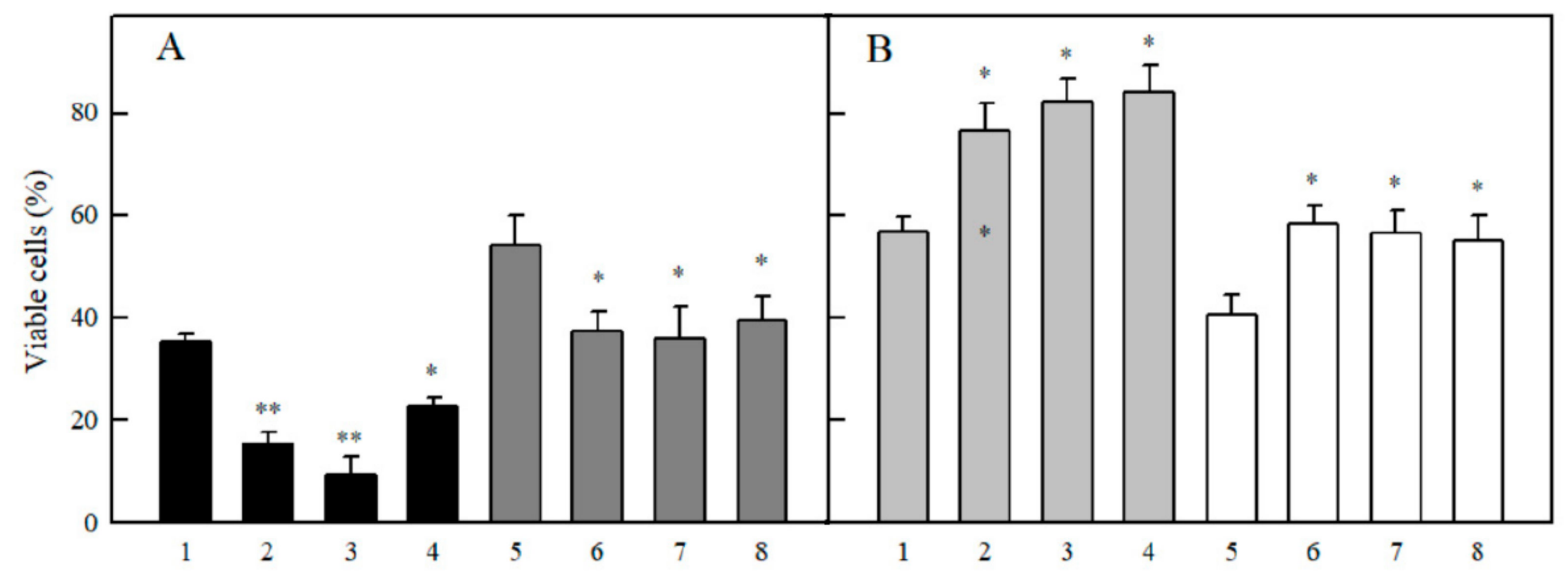
Effects of cytochrome P-450 inhibitors on the cytotoxicity of quinones and ArN→O in MH22a cells. (**A**) Cell viability in the presence of 5.0 µM 9,10-phenanthrene quinone (1–4) and 50 µM tetramethyl-1,4-benzoquinone (5–8), additions: none (1,5), + 5.0 µM α-naphthoflavone (2,6), + 1.0 mM isoniazid (3,7), + 5.0 µM miconazole (4,8). *n* = 3, *p* < 0.01 for 1 against 2,3 (**), *p* < 0.02 for 1 against 4, and for 5 against 6–8 (*). (**B**) Cell viability in the presence of 10 µM 1,2,4-benzotriazine-1,4–dioxide (1-4) and 10 µM 7-F-TPZ (5–8), additions: none (1,5), + 5.0 µM α-naphthoflavone (2,6), + 1.0 mM isoniazid (3,7), + 5.0 µM miconazole (4,8). *n* = 3, *p* < 0.02 for 1 against 2–4, and for 5 against 6–8 (*). Additions of P-450 inhibitors did not affect the cell viability in control experiments, 98.5–99.3%.

The inhibitors of cytochromes P-450 α-naphthoflavone, isoniazide and miconazole enhanced the cytotoxicity of quinones (Figure 7A), and decreased the cytotoxicity of several randomly selected ArN→O (Figure 7B).

## 3. Discussion

The data of our work disclose several properties of heteroaromatic *N-*oxides that are relevant to their cytotoxic/therapeutic action. First, in the context of a limited number of kinetic studies on flavoenzyme-catalyzed reactions involving TPZ derivatives and other ArN→O, our study is the first application of the model of an ‘outer-sphere’ electron transfer for their single-electron reduction (Table 1, Figure 2A,B). Their putative *k*_22_ value, ≥ 10^8^ M^−1^·s^−1^, is consistent with *k*_22_ of quinones and other planar aromatic electron donors, arenes [42]. This may be instrumental in guiding further studies on the activity of new ArN→O against bacteria, viruses, fungi and protozoa [22,23,24], and the prediction of their cytotoxicity/ therapeutic activity towards oxic tumours [21,22] on the basis of their kinetic data.

The most important finding of ours is that in spite of similar redox cycling rates (Figure 2) and lower lipophilicity (Table 3), aromatic *N-*oxides were unexpectedly more cytotoxic than quinones (Figure 4). This phenomenon was observed in two cell lines. Evidently, the observed log (cytotoxicity) vs. *E*^1^_7_ relationships (Figure 4) reflect the superposition of redox cycling caused by single-electron transferring flavoenzymes, and other mechanisms that may oppositely influence the cytotoxicity of ArN→O and quinones.

First, let us discuss the additional mechanisms of quinone cytotoxicity. Although dicoumarol possesses several modes of action in the cell ([43], and references therein), our previous and current data show that in MH22a cells under our experimental conditions, it behaved as an NQO1 inhibitor: it decreased the cytotoxicity of aziridinyl-substituted quinones which stems from NQO1-catalyzed reductive activation [37], and increased the cytotoxicity of tetramethyl-1,4-benzoquinone and 9,10-phenanthrene quinone (Figure 6). In this case, NQO1 converts quinones into relatively stable hydroquinones that can be glucuronated or sulphonated and excreted from the cell ([44], and references therein). Further, it was shown that the inhibition of cytochromes P-450 augmented 2,3-dimethoxy-1,4-naphthoquinone-dependent NADPH oxidation in microsomes, and enhanced its toxicity in hepatocytes [45]. Authors suggest that the inhibition suppresses the electron transfer from P-450R to P-450, and accelerates the reduction of quinones by P-450R. Our data (Figure 7A) are in agreement with this viewpoint.

Concerning the additional mechanisms of cytotoxicity of ArN→O, dicoumarol increased the cytotoxicity of TPZ in hepatocytes under low oxygen pressure, 1% [11]. On the other hand, it protected against TPZ toxicity in A549 cells, besides, their TPZ-resistant subline almost completely lost NQO1 activity [10]. It was shown that oxygen partly inhibits the reductive metabolism of TPZ by isolated NQO1 [9]. In the presence of oxygen, NQO1 oxidized almost a 5-fold excess of NADPH per TPZ, which was explained by the formation of its 4- and 6-electron reduced metabolites [9]. Alternatively, this may also point to the redox cycling of TPZ radicals. Indeed, TPZ stimulated the superoxide dismutase-sensitive reduction of cytochrome *c* by NQO1 (Figure 3), which supports this suggestion. NQO1 reduces quinones and most nitroaromatics in obligatory two-electron way, however, it also performs a single-electron reduction of nitroaromatic compound tetryl [46]. Thus, the dicoumarol protection against the cytotoxicity of TPZ derivatives (Figure 6) may be caused by the inhibition of NQO1-catalyzed redox cycling.

Our finding that cytochrome P-450 inhibitors protect against the cytotoxicity of TPZ derivatives (Figure 7B) is in line with the data on isoniazid protection against TPZ in hepatocytes under low oxygen pressure, 1% [11]. However, the nature of these phenomena is unclear. The role of cytochromes P-450 in the reductive metabolism of TPZ remains not elucidated, it was shown that CO and/or other P-450 inhibitors inhibited TPZ reduction in anaerobic microsomes [12,13] and anaerobic cell sonicates [14], although the absence of influence of CO on microsomal metabolism has also been demonstrated [15]. Besides, P-450 inhibitors metyparone and SKF-525 did not affect TPZ reduction in anaerobic hepatocytes [16], and CO and metyparone did not affect the intensity of ESR signal of TPZ radical in anaerobic microsomes [17]. It has been recently suggested that further progress in this area may be achieved by examining the involvement of P-450 in possible aerobic *N-*oxidation of nor-oxide metabolites of ArN→O [2].

In spite of uncertainty about the mechanism of action of P-450, our data show that NQO1 and P-450 are at least partly responsible for higher cytotoxicity of ArN→O in comparison to quinones. One has to note that there exists another possible but currently poorly understood factor which may enhance the aerobic cytotoxicity of ArN→O. According to recent estimation, during the metabolism of TPZ in aerobic tissue a small amount of DNA-damaging species similar to those forming under hypoxia, may be formed [2]. This side reaction may intensify during the reduction of ArN→O in the nucleus [8], which is poorly oxygenated [47].

Apart from clarifying the mechanisms of action of TPZ and its derivatives in the cell, our data may be helpful in the understanding of the reasons for their relatively high toxicity in vivo, and, possibly, its prediction. According to the scarce available data, TPZ derivatives are more toxic than quinones with similar *E*^1^_7_ values, i.e., similar redox cycling activity. For intraperitoneal toxicity in mice, LD_50_ of 7-Cl-TPZ (*E*^1^_7_ = −0.400 V), TPZ (*E*^1^_7_ = −0.455 V), and 7-CH_3_O- TPZ (*E*^1^_7_ = −0.474 V) are equal to 0.2 mmol/kg, 0.5 mmol/kg, and 1.0 mmol/kg, respectively [48]. In contrast, a similar LD_50_ = 0.09 mmol/kg is characteristic of 2-methyl-1,4-naphthoquinone with *E*^1^_7_ = −0.200 V [49], whereas LD_50_ of 2-hydroxy-1,4-naphthoquinone (*E*^1^_7_ = −0.410 V) is well above 3 mmol/kg [50].

## 4. Materials and Methods

### 4.1. Enzymes and Chemicals

Recombinant rat P-450R, bovine ADR and ADX were prepared as described in [51], their concentrations were determined according to ε_456_ = 21.4 mM^−1^·cm^−1^, ε_450_ = 11.0 mM^−1^·cm^−1^ and ε_414_ = 10.0 mM^−1^·cm^−1^, respectively. NQO1 was prepared from rat liver according to Prochaska [52], its concentration was determined according to ε_460_ = 11.0 mM^−1^·cm^−1^. TPZ and other aromatic *N-*oxides were synthesized as described in [4,18,27]. The compound purity was characterized by IR and NMR spectrometry, melting point, and elemental analysis. NADPH, cytochrome *c*, superoxide dismutase, and other reagents were obtained from Sigma-Aldrich (St. Louis, MO, USA), and used as received.

### 4.2. Enzymatic Assays

The kinetic measurements were carried out spectrophotometrically using a PerkinElmer Lambda 25 spectrophotometer (PerkinElmer, Waltham, MA, USA) in 0.1 M K-phosphate buffer (pH 7.0) containing 1 mM EDTA at 25 °C. The enzyme activities determined according to the rate of reduction of 50 µM cytochrome *c* (∆ε_550_ = 20 mM^−1^·cm^−1^) at substrate concentrations indicated below were close to those reported previously [29,31,34]: 39 s^−1^ (P-450R, [NADPH] = 100 µM), 7.5 s^−1^ (ADR, [ADX] = 0.5 µM, [NADPH] = 50 µM), and 1750 s^−1^ (NQO1, [NADPH] = 150 µM, [menadione] = 10 µM). In this case, 0.01% Tween 20 and 0.25 mg/mL bovine serum albumin were added as NQO1 activators. The initial rates of enzymatic NADPH-dependent *N-*oxide or quinone reduction were determined according to ∆ε_340_ = 6.2 mM^−1^·cm^−1^ after the subtraction of intrinsic NADPH oxidase activities of enzymes, 0.05 s^−1^ (P-450R), 0.1 s^−1^ (NQO1), and 0.11 s^−1^ (ADR + 0.5 µM ADX). The loss of TPZ during its reduction by NQO1 was monitored according to ∆ε_465_ = 6.7 mM^−1^·cm^−1^. Preliminary findings indicate that 1-oxide and nor-oxide metabolites of TPZ do not absorb at this wavelength. The stock solutions of oxidants were prepared in DMSO (dilution factor 100). The values of turnover rate, *k*_cat_, reflecting the maximal number of moles NADPH oxidized or oxidant reduced per mole of the enzyme active center per second, and *k*_cat_/*K*_m_, the bimolecular rate constant (or catalytic efficiency constant), correspond to the inverse intercepts and slopes in Lineweaver-Burk coordinates, [E]/*v* vs. 1/[oxidant]. These rate constants were obtained by fitting the experimental data to the parabolic expression using the SigmaPlot 2000 (version 11.0, Systal Software, San Jose, CA, USA). In some experiments, as noted in the main text, NADPH regeneration system (20 µM NADPH, 10 mM glucose-6-phosphate, and 0.3 mg/mL glucose-6-phosphate dehydrogenase) was used. Oxygen consumption during the reactions was monitored using a Digital Model 10 Clark electrode (rank Brothers Ltd., Bottisham, UK).

### 4.3. Cytotoxicity Assays

Murine hepatoma MH22a cells obtained from Institute of Cytology of the Russian Academy of Sciences (St. Petersburg, Russia), were grown and maintained at 37 °C in DMEM medium, supplemented with 10% fetal bovine serum, 100 U/mL penicillin and 0.1 mg/mL streptomycin, as described in [37]. In the cytotoxicity experiments, 3.0 × 10^4^/mL cells were seeded on 18×18 mm glass slides in 5-mL flasks either in the presence or in the absence of compounds, and were grown for 24 h. In the absence of compounds, cells reached 40–50% confluence. Then, the slides were rinsed 3–4 times with phosphate buffered saline and stained with Trypan blue. The cells adherent to the slides were counted under a light microscope. Typically, they did not accumulate Trypan blue and their viability was 98.5–99.3%. Human colon adenocarcinoma cells HCT-116 obtained from ATCC (Manassas, VA, USA), were grown and maintained at 37 °C in 5% CO_2_ in RPMI 1640 DMEM medium, supplemented with 10% fetal bovine serum, 2 mM l-glutamine and 0.05 mg/mL gentamycin. In the cytotoxicity experiments, 1.0 × 10^5^/mL cells were seeded in the absence or in the presence of compounds, and were grown for 48 h. In the absence of compounds, cells reached 65–75% confluence. Their viability was determined by staining with crystal violet [53]. Stock solutions of compounds were prepared in DMSO. Its concentration in cultivation media did not exceed 0.2%, and did not affect cell viability. The experiments were conducted in triplicate.

### 4.4. Statistical Analysis and Calculations

The statistical analysis was performed using Statistica (version 4.3, Statsoft, Toronto, CA). Octanol/water distribution coefficients at pH 7.0 (log *D*) were calculated using LogD Predictor (https://chemaxon.com).

## Figures and Tables

**Table 1:** The single-electron reduction midpoint potentials (*E*^1^_7_) of compounds and the steady-state bimolecular rate constants (*k*_cat_/*K*_m_) of their reduction by P-450R and ADR/ADX.

No.	Compound	*E*^1^_7_ (V) ^a^	*k*_cat_/*K*_m_ (M^−1^·s^−1^)	
			P-450R	ADR/ADX
	Aromatic *N-*oxides			
1	1,2,4-Benzotriazine-1,4-dioxide	−0.318	4.3 ± 0.4 × 10^5^	4.0 ± 0.3 × 10^5^
2	7-CF_3_-tirapazamine	−0.345	8.7 ± 0.7 × 10^4^	1.9 ± 0.2 × 10^5^
3	7-Cl-tirapazamine	−0.400	6.9 ± 0.7 × 10^4^	7.9 ± 0.6 × 10^5^
4	7-F-tirapazamine	−0.400	3.4 ± 0.3 × 10^4^	5.2 ± 0.6 × 10^5^
5	Tirapazamine	−0.455	1.1 ± 0.1 × 10^4^	1.0 ± 0.1 × 10^5^
6	7-CH_3_-tirapazamine	−0.474	8.6 ± 0.7 × 10^3^	1.2 ± 0.1 × 10^5^
7	7-C_2_H_5_O-tirapazamine	−0.494	4.5 ± 0.5 × 10^3^	1.5 ± 0.1 × 10^5^
8	3-Amino-1,2,4-benzotriazine-1-oxide	−0.568	2.8 ± 0.2 × 10^3^	7.2 ± 0.8 × 10^3^
9	Quinoxaline-1,4-dioxide	−0.575	3.3 ± 0.2 × 10^3^	1.9 ± 0.2 × 10^4^
	Quinones			
10	5-Hydroxy-1,4-naphthoquinone	−0.090	1.5 ± 0.1 × 10^7^	5.6 ± 0.6 × 10^6^
11	5,8-Dihydroxy-1,4-naphthoquinone	−0.110	1.8 ± 0.1 × 10^7^	1.1 ± 0.1 × 10^7^
12	9,10-Phenanthrene quinone	−0.120	1.5 ± 0.2 × 10^7^	6.1 ± 0.5 × 10^6^
13	1,4-Naphthoquinone	−0.150	1.1 ± 0.1 × 10^7^	4.0 ± 0.3 × 10^6^
14	2-Methyl-1,4-naphthoquinone	−0.200	4.2 ± 0.4 × 10^6^	3.6 ± 0.3 × 10^6^
15	Tetramethyl-1,4-benzoquinone	−0.260	8.6 ± 0.6 × 10^5^	9.3 ± 0.7 × 10^5^
16	1,8-Dihydroxy-9,10-anthraquinone	−0.325	1.2 ± 0.3 × 10^5^	6.0 ± 0.7 × 10^5^
17	2-Hydroxy-1,4-naphthoquinone	−0.410	1.3 ± 0.2 × 10^4^	2.0 ± 0.3 × 10^5^

^a^*E*^1^_7_ of aromatic *N-*oxides taken from Ref. [3,17,26], *E*^1^_7_ of quinones taken from Ref. [30].

**Table 2:** The single-electron reduction midpoint potentials (*E*^1^_7_) of aromatic *N-*oxides and the steady-state rate constants of their reduction by NQO1.

No.	Compound	*E*^1^_7_ (V)	*k*_cat_ (s^−1^)	*k*_cat_/*K*_m_ (M^−1^·s^−1^)
1	1,2,4-Benzotriazine-1,4-dioxide	−0.318	1.0 ± 0.10	5.1 ± 0.7 × 10^3^
2	7-CF_3_-tirapazamine	−0.345	1.3 ± 0.10	3.7 ± 0.4 × 10^3^
3	7-Cl-tirapazamine	−0.400	1.0 ± 0.10	4.3 ± 0.4 × 10^3^
4	7-F-tirapazamine	−0.400	0.7 ± 0.10	4.9 ± 0.6 × 10^3^
5	Tirapazamine	−0.455	0.2 ± 0.03	2.4 ± 0.2 × 10^3^
6	7-CH_3_-tirapazamine	−0.474	0.3 ± 0.05	1.7 ± 0.1 × 10^3^
7	7-C_2_H_5_O-tirapazamine	−0.494	0.3 ± 0.04	1.0 ± 0.1 × 10^3^
8	3-Amino-1,2,4-benzotriazine-1-oxide	−0.568	0.1 ± 0.02	1.6 ± 0.2 × 10^3^
9	Quinoxaline-1,4-dioxide	−0.575	0.6 ± 0.10	7.9 ± 0.9 × 10^3^

**Table 3:** The single-electron reduction midpoint potentials (*E*^1^_7_), octanol/water distribution coefficients at pH 7.0 (log *D*) of compounds, their concentrations for 50% cell survival (cL_50_) in murine hepatoma MH22a cells, and in their concentrations causing 50% maximal proliferation inhibition (GI_50_) of human colon adenocarcinoma HCT-116 cells.

No.	Compound	*E*^1^_7_ (V)	log *D*	cL_50_ (µM) MH22a	GI_50_ (µM) HCT-116
	Aromatic *N-*oxides				
1	1,2,4-Benzotriazine-1,4-dioxide	−0.318	−0.70	11 ± 1.5	n.d.
2	7-CF_3_-tirapazamine	−0.345	0.76	3.4 ± 0.4	6.0 ± 1.0
3	7-Cl-tirapazamine	−0.400	0.49	3.1 ± 0.5	13 ± 1.5
4	7-F-tirapazamine	−0.400	0.03	7.2 ± 1.0	n.d.
5	Tirapazamine	−0.455	0.11	31 ± 5.5	75 ± 7.0
6	7-CH_3_-tirapazamine	−0.474	0.40	83 ± 10	50 ± 6.0
7	7-C_2_H_5_O-tirapazamine	−0.494	0.08	64 ± 10	60 ± 7.0
8	3-Amino-1,2,4-benzotriazine-1-oxide	−0.568	0.30	≥ 600	≥ 600
9	Quinoxaline-1,4-dioxide	−0.575	−0.90	325 ± 40	n.d.
	Quinones				
10	5-Hydroxy-1,4-naphthoquinone	−0.090	1.82	2.5 ± 0.04 ^a^	0.30 ± 0.05
11	5,8-Dihydroxy-1,4-naphthoquinone	−0.110	2.19	0.58 ± 0.05 ^a^	0.25 ± 0.04
12	9,10-Phenanthrene quinone	−0.120	2.92	4.6 ±0.3 ^a^ 3.9 ± 0.4 ^b^	1.0 ± 0.1
13	1,4-Naphthoquinone	−0.150	1.49	3.1 ±0.05 ^a^	1.0 ± 0.1
14	2-Methyl-1,4-naphthoquinone	−0.200	1.89	18 ± 1.3 ^a^	7.0 ± 1.2
15	Tetramethyl-1,4-benzoquinone	−0.260	2.61	59 ± 5.0 ^a^ 50 ± 5.0 ^b^	45 ± 6.5
16	1,8-Dihydroxy-9,10-anthraquinone	−0.325	3.56	120 ± 15 ^a^	n.d.
17	2-Hydroxy-1,4-naphthoquinone	−0.410	−0.52	500 ± 80 ^a^ 430 ± 60 ^b^	300 ± 28

^a^ Taken from Ref. [37]. ^b^ This work.

## Abbreviations

ADR	NADPH:adrenodoxin reductase
ADX	Adrenodoxin
ArN→O	Heteroaromatic *N-*oxide
BCNU	1,3-bis(2-chloroethyl)-1-nitrosourea
cL_50_	Concentration for 50% cell survival
DPPD	*N,N’-*diphenyl-*p-*phenylene diamine
*E* ^1^_7_	Single-electron reduction midpoint potential
GI_50_	Concentration for 50% inhibition of maximal cell proliferation
*k*_cat_	Catalytic constant
*k*_cat_/*K*_m_	Bimolecular rate constant
LD_50_	Median lethal dose
log *D*	Octanol/water distribution coefficient at pH 7.0
NQO1	NAD(P)H:quinone oxidoreductase
P-450	Cytochrome P-450
P-450R	NADPH:cytochrome P-450 reductase
TPZ	Tirapazamine

## Appendix A

Calculation of electron self-exchange rate constant for heteroaromatic *N-*oxides

At ∆*E*^1^ = 0, Equation (1) may be expressed as

log *k*_12_ = (log *k*_11_ + log *k*_22_)/2 (3)

For the calculation of electron self-exchange rate constant for heteroaromatic *N-*oxides, we use the rate constants of oxidation of radicals of 3,6,7-substituted 2-carboxy-4′-methylpiperazide- quinoxaline-1,4-dioxides by oxygen [5]. The reactivity of compounds decreases with their *E*^1^ values, and is described by Equation (4):

log *k*_12_ = (4.92 ± 0.15) – (4.52 ± 0.31) × *E*^1^ (4)

According to Equation (4), at ∆*E*^1^ = 0, i.e., at *E*^1^ being equal to −0.155 V (midpoint potential of O_2_/O_2_^−·^ couple), log *k*_12_ = 5.62. Using log *k*_22_ = 2.65 for O_2_/O_2_^·−^ couple [33], we obtain log *k*_11_ = 8.59 for aromatic *N-*oxides.
